# Supplementary material for: Determinants of Campylobacter species diversity in infants and association with family members, livestock, and household environments in rural Eastern Ethiopia
Source: Res Sq. 2025 Jan 15:rs.3.rs-5672139. Preprint. [Version 1] doi: 10.21203/rs.3.rs-5672139/v1 (PMC11774460; doi:10.21203/rs.3.rs-5672139/v1)
Supplement: Supplement 1 [file NIHPPrs5672139v1-supplement-1.pdf]

## Supplementary Files

This is a list of supplementary files associated with this preprint. Click to download.

- Cagedsppsupplemental121724.pdf
